# IncX4 Plasmid Carrying the New *mcr-1.9* Gene Variant in a CTX-M-8-Producing *Escherichia coli* Isolate Recovered From Swine

**DOI:** 10.3389/fmicb.2019.00367

**Published:** 2019-03-14

**Authors:** Vera Manageiro, Lurdes Clemente, Raquel Romão, Catarina Silva, Luís Vieira, Eugénia Ferreira, Manuela Caniça

**Affiliations:** ^1^National Reference Laboratory of Antibiotic Resistances and Healthcare Associated Infections, Department of Infectious Diseases, National Institute of Health Dr. Ricardo Jorge, Lisbon, Portugal; ^2^Centre for the Studies of Animal Science, Institute of Agrarian and Agri-Food Sciences and Technologies, University of Porto, Porto, Portugal; ^3^Bacteriology and Mycology Laboratory, INIAV – National Institute of Agrarian and Veterinary Research, Oeiras, Portugal; ^4^Innovation and Technology Unit, Department of Human Genetics, National Institute of Health Dr. Ricardo Jorge, Lisbon, Portugal

**Keywords:** MCR-1.9, plasmid-mediated colistin resistance, IncX4, CTX-M-8, Portugal

## Abstract

We studied a commensal colistin-resistant *Escherichia coli* isolated from a swine cecum sample collected at a slaughter, in Portugal. Antimicrobial susceptibility phenotype of *E. coli* LV23529 showed resistance to colistin at a minimum inhibitory concentration of 4 mg/L. Whole genome of *E. coli* LV23529 was sequenced using a MiSeq system and the assembled contigs were analyzed for the presence of antibiotic resistance and plasmid replicon types using bioinformatics tools. We report a novel *mcr-1* gene variant (*mcr-1.9*), carried by an IncX4 plasmid, where one-point mutation at nucleotide T1238C leads to Val413Ala substitution. The *mcr-1.9* genetic context was characterized by an IS*26* element upstream of the *mcr-pap2* element and by the absence of IS*Apl1*. Bioinformatic analysis also revealed genes conferring resistance to β-lactams, sulphamethoxazole, trimethoprim, chloramphenicol and colistin, corresponding to the phenotype noticed. Moreover, we highlight the presence of *mcr-1.9* plus *bla*_CTX-M-8_, a *bla*_ESBL_ gene rarely detected in Europe in isolates of animal origin; these two genes were located on different plasmids with 33,303 and 89,458 bp, respectively. MCR-1.9-harboring plasmid showed high identity to other X4-type *mcr-1*-harboring plasmids characterized worldwide, which strongly suggests that the presence of PMCR-encoding genes in food-producing animals, such as MCR-1.9, represent a potential threat to humans, as it is located in mobile genetic elements that have the potential to spread horizontally.

## Introduction

Since the report of a plasmid-mediated colistin resistance (PMCR) mechanism, designated MCR-1, in *Escherichia coli* and *Klebsiella pneumoniae* isolated from animals, food and humans in China, further reports exposed the global dissemination of *mcr*-type gene in various bacterial species isolated from a wide range of different sources (Caniaux et al., 2017). In Portugal, PMCR has also been detected in a wide range of different sources and species, including humans, food-producing animals and meat, and in the environment (Campos et al., 2016; Figueiredo et al., 2016; Jones-Dias et al., 2016; Kieffer et al., 2017; Manageiro et al., 2017; Tacão et al., 2017; Mendes et al., 2018). Noteworthy, are the recent report of two cases presumably associated with the travel of patients from Portugal, one involving animals: a patient repatriated to France after hospitalization for 2 months in Portugal, in 2015 (Beyrouthy et al., 2017), and a New York state patient returning from Portugal in 2016 after staying on a farm with chickens and pigs (Gilrane et al., 2017).

More worrisome is the presence of *mcr* genes in *Enterobacteriaceae* carrying other resistance determinants namely, extended-spectrum β-lactamases (ESBL)- and/or carbapenemase-encoding genes. Since the first report of co-localization of *mcr-1* and ESBL- in 2016 in bovines in France, an increase encoding genes in the proportion of *mcr-1* genes among ESBL-producing *E. coli* in animals has been noticed, suggesting that the use of extended-spectrum cephalosporins may have simultaneously favored the spread of *mcr-1* (Haenni et al., 2016). Here we describe the first detection of a novel *mcr* variant, hereafter-named *mcr-1.9*, identified in a commensal *E. coli* LV23529 isolated from a swine cecum sample collected at a slaughter, in Portugal.

## Materials and Methods

### Bacterial Isolate

*Escherichia coli* LV23529 was isolated in 2015 from a swine cecum sample collected at a Portuguese slaughter, during an evaluation study of commensal *E. coli* recovered from swine samples for antimicrobial susceptibility testing.

### Antimicrobial Susceptibility Testing

Minimum inhibitory concentrations (MICs) were determined by microdilution method as previously described (Manageiro et al., 2017). In order to assess decreased susceptibility of the strain, interpretation of the results was done according to the epidemiological cut-off values recommended by the European Committee on Antimicrobial Susceptibility Testing (EUCAST^1^).

### Screening and Characterization of PMCR- and ESBL-Resistance Mechanisms

#### Molecular Detection of *mcr-1* and *bla*_ESBL_-Encoding Genes

Following phenotypic characteristics, PMCR- and ESBL-resistance mechanisms were searched and identified by molecular methods, as previously described (Manageiro et al., 2017).

#### Transfer Experiments

Conjugation experiments were performed using sodium azide-resistant *E. coli* J53 as a recipient strain. Transconjugants were selected on McConkey agar supplemented with sodium azide (150 mg/L) and either cefotaxime (2 mg/L) or colistin (2 mg/L). Plasmid DNA was extracted from *E. coli* LV23529 using a NucleoBond Xtra Plus kit (Macherey-Nagel), and transformed into *E. coli* TOP10 OneShot chemically competent cells (Invitrogen), accordingly to manufacture’s protocol. *E. coli* transformants were selected on MacConkey agar supplemented with 2 mg/L of colistin. PCR for *bla*_CTX-M-8_ or *mcr-1*-type and MICs of recipients and transformants were determined as mentioned above.

#### Genetic Context of *mcr-1.9* Gene

Colistin-resistant *E. coli* LV23529 was genotypically characterized by whole-genome sequencing (WGS), as previously described (Manageiro et al., 2017). Sequence reads were trimmed and filtered according to quality criteria, and de novo assembled into contigs by means of CLC Genomics Workbench 10.0 (Qiagen). The assembled contigs were analyzed and studied for the presence of antibiotic resistance, virulence genes and plasmid replicon types, serotype, multi-locus sequence type (ST) and *fim*-type, using bioinformatics tools^2^. The NCBI prokaryotic genome automatic annotation pipeline (PGAAP) was used for annotation.

Plasmid sequencing was also performed on a MiSeq Illumina platform using 150 bp paired-end reads, after plasmid DNA extraction from TLV23529 (*mcr-1.9*) using a NucleoBond Xtra Plus kit (Macherey-Nagel), and quantification using Qubit 1.0 Fluorometer (Invitrogen), as previously described (Manageiro et al., 2017). Sequence reads were trimmed and filtered according to quality criteria, and mapped against *E. coli* ATCC 25922 genome (NZ_CP009073). Unmapped reads (80.2%/total reads) were then used for plasmids structure construction by mapping assembly based on the genetic organization of the closest plasmid sequences obtained by BLASTn; this was followed by contig neighbor’s prediction from assembly information using CLC Genomics Workbench 10.0 (Qiagen). NCBI Microbial genomes BLAST analysis tool^3^ was used to search for plasmid sequences. Plasmid alignments and ORF representations were also done using EasyFig v. 2.2.3 (Sullivan et al., 2011).

#### Genomic Epidemiological Analysis

BacWGSTdb database was used for genotyping and source tracking bacterial pathogen (Ruan and Feng, 2016).

#### Nucleotide Sequence Accession Number

The pLV23529-MCR-1.9 and pLV23529-CTX-M-8 nucleotide sequences from this study were submitted to the NCBI GenBank Database with accession numbers KY964067 and KY964068, respectively. The new *mcr-1.9* nucleotide sequence was submitted with accession number KY780959.

This Whole Genome Shotgun project has been deposited at DDBJ/ENA/GenBank under the accession SBIH00000000. The version described in this paper is version SBIH01000000.

## Results and Discussion

MIC results showed that LV23529 was non-wild-type to third- and fourth-generation cephalosporins (ceftazidime 2 mg/L, cefotaxime 32 mg/L, cefepime 8 mg/L) with synergy with clavulanic acid; this isolate was also non-wild-type to chloramphenicol (>128 mg/L), sulphamethoxazole (>1024 mg/L), trimethoprim (>32 mg/L), tetracycline (>64 mg/L), and colistin (4 mg/L). LV23529 remained wild-type to carbapenems, fluoroquinolones, aminoglycosides and tigecycline (Table 1).

**Table 1:** Phenotypic and genotypic context of CTX-M-8 and MCR-1.9 producing *E. coli* clinical isolate, transformant, transconjugant, and the respective recipient strains.

Antibiotic	LV23529^b^	Transformation		Conjugation	
	(*bla*_CTX-M-8_, *mcr-1.9*, *cmlA1*, *sul3*, *tetA*, *tetM*, *dfrA12*, *aadA1*, *aadA2*)	*E. coli* TOP10^c^	TLV23529^d^ (*mcr-1.9*)	ECJ53AZNa^e^	TcLV2352^f^ (*bla*_CTX-M-8_)
Ampicillin	>64	4	8	2	>64
Cefoxitine	4	4	4	4	8
Ceftazidime	2	0.5	0.5	≤0.5	1
Ceftazidime plus clavulanate^a^	≤0.125/4	0.5	0.5	≤0.125/4	≤0.25/4
Cefotaxime	32	≤0.25	≤0.25	≤0.25	2
Cefotaxime plus clavulanate^a^	≤0.06/4	0.125	0.125	≤0.06/4	≤0.06/4
Cefepime	8	0.125	0.125	≤0.06	2
Imipenem	0.25	0.5	0.5	0.25	0.25
Meropenem	≤0.03	0.06	0.06	≤0.03	≤0.03
Ertapenem	≤0.015	≤0.015	≤0.015	≤0.015	≤ 0.015
Nalidixic acid	≤4	≤4	≤4	≤4	≤4
Ciprofloxacin	≤0.015	≤0.015	≤0.015	≤0.015	≤0.015
Chloramphenicol	>128	≤8	≤8	≤8	≤8
Sulphamethoxazole	>1024	≤8	≤8	≤8	≤8
Tetracycline	>64	≤2	≤2	≤2	≤2
Trimethoprim	>32	≤0.25	≤0.25	≤0.25	≤0.25
Gentamicin	≤0.5	≤0.5	≤0.5	≤0.5	≤0.5
Colistin	4	≤1	2	≤1	≤1
Tigecycline	≤0.25	≤0.25	≤0.25	≤0.25	≤0.25


*MICs in mg/L.*

*^*a*^Clavulanate 4 mg/L.*

*^*b*^*E. coli* LV23529 was the clinical isolate harboring the acquired antibiotic resistance genes *bla*_*CTX-M-8*_, *mcr-1.9*, *cmlA1*, *sul3*, *tetA*, *tetM*, *dfrA12*, *aadA1*, and *aadA2*.*

*^*c*^*E. coli* TOP10 was the recipient strain in the transformation experiment.*

*^*d*^TLV23529 is a transformant of LV23529 (harboring *mcr-1.9*).*

*^*e*^*E. coli* J53AZNa was the recipient strain in the conjugation experiment.*

*^*f*^TcLV23529 is a transconjugant of LV23529 (harboring *bla*_*CTX-M-8*_).*

Molecular characterization of the *E. coli* LV23529 isolate allowed the detection of *bla*_CTX-M-8_ and *mcr-1*-type genes.

Only the transferability of the *bla*_CTX-M-8_ gene was achieved by conjugation, with TcLV23529 (*bla*_CTX-M-8_) exhibiting the ESBL phenotype from LV23529 isolate (cefotaxime 2 mg/L, cefepime 2 mg/L) with synergy with clavulanic acid, and wild-type to colistin (≤1 mg/L) (Table 1). Although conjugation assays for *mcr-1*-type were negative, the colistin resistance determinant could be transferred to *E. coli* TOP10 competent cells; transformant TLV23529 (*mcr-1*-type) showed the respective resistance to colistin (4 mg/L) (Table 1).

The WGS assembly of *E. coli* LV23529 yielded 193 contigs (average 143.7-fold coverage), which together comprised 5,122,415bp, showing a GC content of 50.7%. The largest contig was 320,931 bp long; the N50 statistic, which stands for the minimum contig length of at least 50% of the contigs, was 113,197 bp. The average length of the obtained contigs was 26,541 bp. Overall, the genome sequence comprised 5,124 putative genes, among which 5,037 consisted of protein encoding sequences.

The WGS analysis showed that *E. coli* LV23529 belongs to serotype O8:H19, usually associated with porcine *stx*_2e_-producing *E. coli* (Zweifel et al., 2006; Bai et al., 2015), and to MLST (Achtman scheme) ST201 [clonal complex 469 (CC469)] and to the FimH-type determinant *fimH32*. This ST201 was encountered worldwide mainly in isolates collected from livestock samples (*Escherichia*/*Shigella* Enterobase database, Alikhan et al., 2018). Three virulence factors were detected: *astA* (heat-stable enterotoxin 1), *lpfA* (long polar fimbriae), and *gad*-type (glutamate decarboxylase).

Further bioinformatics analysis of *E. coli* LV23529 isolate revealed acquired-genes conferring resistance to β-lactams (*bla*_CTX-M-8_ and *bla*_TEM-1_), aminoglycosides (*aadA1* and *aadA2*), phenicol (*cmlA1-type* and *floR*-type), sulphamethoxazole (*sul3*), tetracycline [*tet(A)*-type and *tet(M)*-type], trimethoprim (*dfrA12*), and colistin (*mcr-1-type*), justifying the phenotype noticed. Additionally, several unknown mutations in the *ampC* (promoter region), *parC*, 16S *rrsB*, 16S *rrsC*, 23S and *pmrB* chromosomal genes were detected, the last gene being described as the primary mechanism for the development of chromosomally encoded resistance to polymyxins (Phan et al., 2017).

The named *mcr-1.9*, differed from *mcr-1* by one-point mutation (T1238C), leading to Val413Ala substitution. The MCR-1 protein contains a transmembrane domain and a phosphoethanolamine (PEA) transferase domain with 8α, 12β, and 12η units (Gao et al., 2016). The amino acid substitution of MCR-1.9 occurred in the region between η7 e η8 of the PEA transferase domain, which have been found not to influence the function of MCR-1 (Gao et al., 2016).

**FIGURE 1:**
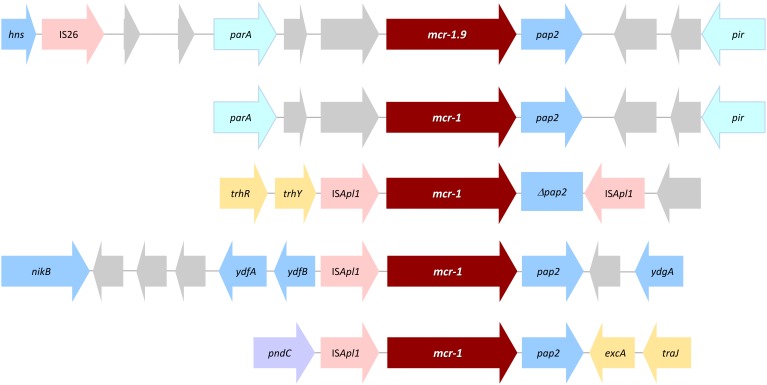
Linear comparison of IncX4-pLV23529-MCR-1.9 with the top six *mcr-1*-harboring plasmids showing the highest identities (>99.9%, E-value 0.0), in different *E. coli* isolates. Boxed arrows represent the position and transcriptional direction of ORFs. Gray vertical blocks indicate the shared similarity regions according to TBLASTX identity. Genes associated with pilus and plasmid transfer are colored yellow, antibiotic resistance genes are colored red, mobile genetic elements are colored pink, and other genes are colored gray (hypothetical proteins) or blue (other).

The *mcr-1.9* genetic context was characterized by an IS*26* element upstream of the *mcr-pap2* element and by the absence of IS*Apl1* (Figure 1), which is in accordance with other studies about *mcr-1* gene (Veldman et al., 2016; Sun et al., 2017). The *mcr-1*.9 gene can be mobilized within an IS*Apl1*-flanked composite transposon (Tn*6330*), although many sequences have been identified without IS*Apl1* or with just a single copy (Snesrud et al., 2018). Indeed, it has been described that initially IS*Apl1* was presumably involved in the transposition of the *mcr-1* cassette and then was lost, contributing for the stability of *mcr* gene on IncX4 plasmids (Sun et al., 2017; Snesrud et al., 2018).

**FIGURE 2:**
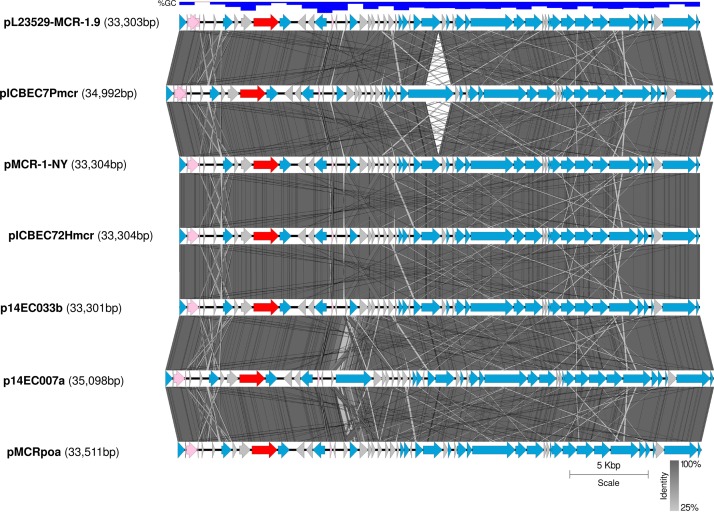
Schematic representation of the genetic environment of *mcr-1.9* in comparison with other *mcr-1*-type representative environments. Boxed arrows represent the position and transcriptional direction of ORFs. Genes are not drawn to scale. Genes associated with pilus and plasmid transfer are colored yellow, antibiotic resistance genes are in red, mobile genetic elements in pink, plasmid maintenance and stability genes in violet, plasmid replication associated genes are in light blue, and other genes are colored gray (hypothetical proteins) or blue (other).

The PMCR-encoding gene was found in an IncX4 plasmid (pLV23529-MCR-1.9), showing highest identities (>99.9%) with six IncX4-type *mcr-1*-harboring plasmids identified worldwide, in unrelated *E. coli* isolates, mainly collected from human patients (Figure 2 and Table 2). Indeed, all belonged to different MLST, which might suggest a resistance plasmid dissemination across strains (plasmid outbreak) rather than clonal transmission of MCR-1-type-producing strains. Furthermore, no *E. coli* LV23529 closely related isolates were detected among those currently deposited in the public database BacWGSTdb (Ruan and Feng, 2016), which reinforce the importance of the horizontal gene transfer in this study.

**Table 2:** Comparison of IncX4-pLV23529-MCR-1.9 with the top six *mcr-1*-harboring plasmids showing the highest identities (>99.9%, E-value 0.0), in different *E. coli* isolates.

IncX4-type Plasmid (bp)	*E. coli* strain (MLST^a^)	Source/Country/Year	Identity (%)	Mismatches/gap opens (No. of nucleotides)	Query alignment overlap (%)	pLV23529-MCR-1.9 Alignment overlap (%)	Plasmid GenBank Acc. No.
**pMCRpoa** (33,511)	3431F (ST744)	Human patient/Brazil/2014	99.98	**15/1**	99.4	100.0	CM007714
**pl4EC007a** (35,098)	14Ec007 (ST301)	Human patient/China/2014	99.98	**15/1**	94.9	100.0	CP024132
**pMCR-1-NY** (33,304)	MDR56 (ST117)	Human patient /United States/2015	99.97	**19/1**	100.0	100.0	CP019908
**pICBEC72Hmcr** (33,304)	ICBEC72H (ST101)	Human patient/Brazil/2016	99.95	**17/0**	100.0	100.0	CP015977
**pl4EC033b** (33,301)	14EC033 (ST2064)	Human patient/China/2014	99.92	**16/3**	100.0	100.0	CP024149
**pICBEC7Pmcr** (34,992)	ICBEC7P (ST10)	Magellanic penguins/Brazil/2013	99.92	**16/3**	95.2	100.0	CPO17246


*^*a*^MLST accordingly with Warwick scheme (http://mlst.warwick.ac.uk/mlst/dbs/Ecoli).*

**FIGURE 3:**
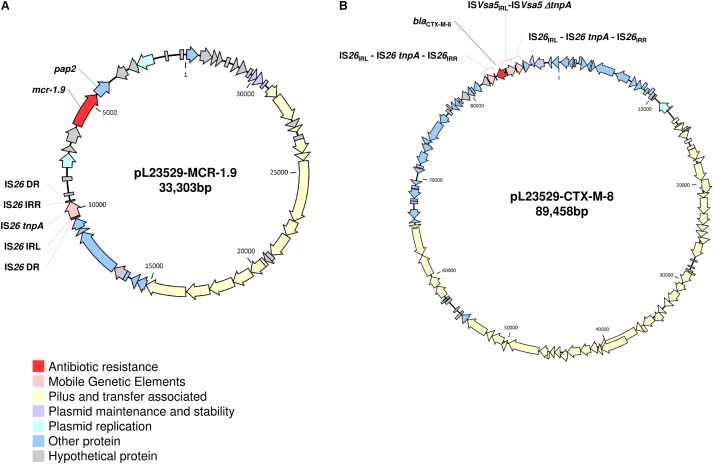
Schematic maps of pLV23529-MCR-1.9 **(A)** and pLV23529-CTX-M-8 **(B)**. Genes are denoted by arrows and colored based on gene function classification.

Like pLV23529-MCR-1.9, the six plasmids (Table 2) doesn’t have the IS*Apl1* element. Hence, similarities may suggest that the one-point mutation (T1238C) in *mcr-1.9* occurred on the X4 plasmid, since mobilization of *mcr-1* occurs as part of a composite transposon (Tn*6330*) and that structures lacking the downstream IS*Apl1* are not capable of mobilization (Snesrud et al., 2018). The IS*26* upstream of the *mcr-pap2* element is flanked by an 8bp direct repeat (Figure 3A), indicating that its insertion wouldn’t seems to be related to the *mcr-1.9* context, justifying the differences found with other IncX4 *mcr-1*-harboring plasmids. IncX4 plasmid has been widely implicated in the spread of MCR-1 gene in Europe (Caniaux et al., 2017). In Portugal, this plasmid type is circulating among diverse hosts (humans, pigs, poultry), being responsible for hospital-based outbreak caused by MCR-1 plus KPC-3-producing *K. pneumoniae* (Mendes et al., 2018), as well as for the diffusion of this PMCR at the farm level (Kieffer et al., 2017). Indeed, IncX4 plasmids seem to be efficiently transferred at different temperatures and different lack-of-fitness burdens among bacterial hosts, which may facilitate the transfer of *mcr-type* among *Enterobacteriaceae* (Lo et al., 2014; Wu R. et al., 2018). The pLV23529-MCR-1.9 plasmid backbone contains all the core genes common to IncX plasmids involved in segregation, stability, replication, and conjugative transfer of the plasmid (Figure 3A), namely the IncX-type pilus synthesis operon (*pilX1*-*pilX11*). However, pLV23529-MCR-1.9 was mobilizable, but not self-transmissible. Of note, we found a one-point mutation (G64T), leading to Asp22Tyr substitution, in the PilX1, a peptidoglycan hydrolase involved in T-DNA plasmid transfer. This mutation might explain why the attempts to conjugate *mcr-1.9* from *E. coli* LV23529 were unsuccessful (Chen et al., 2009).

Further plasmid analysis revealed the presence of two other plasmids: IncF [F2:A-:B-], IncR and the colicinogenic IncI1-ST113-carrying the *bla*_CTX-M-8_ (pLV23529-CTX-M-8, Figure 3B). Of note, the *mcr-1.9*-positive isolate, co-harboring *bla*_CTX-M-8_ and *bla*_TEM-1_ genes, is here reported for the first time in an *E. coli* isolate of animal origin. In fact, *bla*_CTX-M-8_ gene is rarely detected in Europe in isolates of animal origin (Börjesson et al., 2016), but in humans seems to be emerging (Eller et al., 2014). Indeed, a recent phylogenetic study suggested an increasing trend of co-existence and transmission of *bla*_CTX-M_ and *mcr-1* in both clinical medicine and veterinary medicine (Wu C. et al., 2018).

In conclusion, the presence of PMCR-encoding genes, such as MCR-1.9, in food-producing animals represents a potential threat to humans, as it is located in mobile genetic elements that have the potential to spread horizontally. As mentioned, in Portugal, PMCR is an emerging problem and its international spread is a worrying reality (Beyrouthy et al., 2017; Gilrane et al., 2017).

## Author Contributions

VM designed the study, performed the molecular experiments and bioinformatics analysis, interpreted the data, and wrote the manuscript. LC, RR, and EF performed the microbiological and molecular experiments. CS and LV performed the Illumina genome sequencing experiments. MC designed the study, wrote, reviewed and edited the manuscript. All authors read and approved the final manuscript.

## Conflict of Interest Statement

The authors declare that the research was conducted in the absence of any commercial or financial relationships that could be construed as a potential conflict of interest.

## Footnotes

1 http://mic.eucast.org/Eucast2/

2 https://cge.cbs.dtu.dk/services/

3 https://www.ncbi.nlm.nih.gov/genome/microbes/

## References

[B1] Alikhan N. F. Zhou Z. Sergeant M. J. Achtman M. (2018). A genomic overview of the population structure of *Salmonella*. *PLoS Genet.* 14:e1007261. 10.1371/journal.pgen.1007261 29621240 PMC5886390

[B2] Bai X. Wang H. Xin Y. Wei R. Tang X. Zhao A. (2015). Prevalence and characteristics of Shiga toxin-producing *Escherichia coli* isolated from retail raw meats in China. *Int. J. Food Microbiol.* 200 31–38. 10.1016/j.ijfoodmicro.2015.01.018 25676240

[B3] Beyrouthy R. Robin F. Lessene A. Lacombat I. Dortet L. Naas T. (2017). MCR-1 and OXA-48 in vivo acquisition in KPC-producing *Escherichia coli* after colistin treatment. *Antimicrob. Agents Chemother.* 61:e02540-16. 10.1128/AAC.02540-16 28507112 PMC5527634

[B4] Börjesson S. Ny S. Egervärn M. Bergström J. Rosengren Å Englund S. (2016). Limited dissemination of extended-spectrum β-lactamase- and plasmid-encoded Ampc-producing *Escherichia coli* from food and farm animals, Sweden. *Emerg. Infect. Dis.* 22 634–640. 10.3201/eid2204.151142 26982890 PMC4806949

[B5] Campos J. Cristino L. Peixe L. Antunes P. (2016). MCR-1 in multidrug-resistant and copper-tolerant clinically relevant *Salmonella* 1,4,[5],12:i:- and S. Rissen clones in Portugal, 2011 to 2015. *Euro Surveill.* 21:30270. 10.2807/1560-7917.ES.2016.21.26.30270 27387036

[B6] Caniaux I. van Belkum A. Zambardi G. Poirel L. Gros M. F. (2017). MCR: modern colistin resistance. *Eur. J. Clin. Microbiol. Infect.* 36 415–420. 10.1007/s10096-016-2846-y 27873028

[B7] Chen C.-L. Wang C.-Y. Chu C. Su L.-H. Chiu C.-H. (2009). Functional and molecular characterization of pSE34 encoding a type IV secretion system in *Salmonella enterica* serotype Enteritidis phage type 34. *FEMS Immunol. Med. Microbiol.* 5 274–283. 10.1111/j.1574-695X.2009.00612.x 19817860

[B8] Eller C. Leistner R. Guerra B. Fischer J. Wendt C. Rabsch W. (2014). Emergence of extended-spectrum β-lactamase (ESBL) CTX-M-8 in Germany. *J. Antimicrob. Chemother.* 69 562–564. 10.1093/jac/dkt387 24072171

[B9] Figueiredo R. Card R. M. Nunez J. Pomba C. Mendonça N. Anjum M. F. (2016). Detection of an *mcr*-1-encoding plasmid mediating colistin resistance in *Salmonella enterica* from retail meat in Portugal. *J. Antimicrob. Chemother.* 71 2338–2340. 10.1093/jac/dkw240 27330063

[B10] Gao R. Hu Y. Li Z. Sun J. Wang Q. Lin J. (2016). Dissemination and mechanism for the MCR-1 colistin resistance. *PLoS Pathog.* 12:e1005957. 10.1371/journal.ppat.1005957 27893854 PMC5125707

[B11] Gilrane V. L. Lobo S. Huang W. Zhuge J. Yin C. Chen D. (2017). Complete genome sequence of a colistin-resistant *Escherichia coli* strain harboring *mcr-1* on an IncHI2 plasmid in the United States. *Genome Announc.* 5:e01095-17. 10.1128/genomeA.01095-17 29051246 PMC5646399

[B12] Haenni M. Métayer V. Gay E. Madec J. Y. (2016). Increasing trends in *mcr-1* prevalence among extended-spectrum-β-lactamase-producing *Escherichia coli* isolates from french calves despite decreasing exposure to colistin. *Antimicrob. Agents Chemother.* 60 6433–6434. 10.1128/AAC.01147-16 27503658 PMC5038315

[B13] Jones-Dias D. Manageiro V. Ferreira E. Barreiro P. Vieira L. Moura I. B. (2016). Architecture of class 1, 2, and 3 integrons from Gram negative bacteria recovered among fruits and vegetables. *Front. Microbiol.* 7:1400. 10.3389/fmicb.2016.01400 27679611 PMC5020092

[B14] Kieffer N. Aires-de-Sousa M. Nordmann P. Poirel L. (2017). High rate of MCR-1-producing *Escherichia coli* and *Klebsiella pneumoniae* among pigs. *Portugal. Emerg. Infect. Dis.* 23 2023–2029. 10.3201/eid2312.170883 29148380 PMC5708242

[B15] Lo W. U. Chow K. H. Law P. Y. Ng K. Y. Cheung Y. Y. Lai E. L. (2014). Highly conjugative IncX4 plasmids carrying *bla*_CTX-M_ in *Escherichia coli* from humans and food animals. *J. Med. Microbiol.* 63 835–840. 10.1099/jmm.0.074021-0 24595536

[B16] Manageiro V. Clemente L. Graça R. Correia I. Albuquerque T. Ferreira E. (2017). New insights into resistance to colistin and third-generation cephalosporins of *Escherichia coli* in poultry, Portugal: novel *bla*_CTX-M-166_ and *bla*_ESAC_ genes. *Int. J. Food Microbiol.* 263 67–73. 10.1016/j.ijfoodmicro.2017.10.007 29031106

[B17] Mendes A. C. Novais Â Campos J. Rodrigues C. Santos C. Antunes P. (2018). *mcr-1* in carbapenemase-producing *Klebsiella pneumoniae* with hospitalized patients, Portugal, 2016-2017. *Emerg. Infect. Dis.* 24 762–766. 10.3201/eid2404.171787 29553327 PMC5875258

[B18] Phan M. D. Nhu N. T. K. Achard M. E. S. Forde B. M. Hong K. W. Chong T. M. (2017). Modifications in the pmrB gene are the primary mechanism for the development of chromosomally encoded resistance to polymyxins in uropathogenic *Escherichia coli*. *J. Antimicrob. Chemother.* 72 2729–2736. 10.1093/jac/dkx204 29091192

[B19] Ruan Z. Feng Y. (2016). BacWGSTdb, a database for genotyping and source tracking bacterial pathogens. *Nucleic Acids Res.* 44 D682–D687. 10.1093/nar/gkv1004 26433226 PMC4702769

[B20] Snesrud E. McGann P. Chandle M. (2018). The birth and demise of the IS*Apl1*-*mcr*-*1*-IS*Apl1* composite transposon: the vehicle for transferable colistin resistance. *mBio* 9:e02381-17. 10.1128/mBio.02381-17 29440577 PMC5821093

[B21] Sullivan M. J. Petty N. K. Beatson S. A. (2011). Easyfig: a genome comparison visualizer. *Bioinformatics* 27 1009–1010. 10.1093/bioinformatics/btr039 21278367 PMC3065679

[B22] Sun J. Fang L. X. Wu Z. Deng H. Yang R. S. Li X. P. (2017). Genetic analysis of the IncX4 plasmids: implications for a unique pattern in the *mcr-1* acquisition. *Sci. Rep.* 7:424. 10.1038/s41598-017-00095-x 28336940 PMC5428312

[B23] Tacão M. Tavares R. D. S. Teixeira P. Roxo I. Ramalheira E. Ferreira S. (2017). *mcr-1* and *bla*_KPC-3_ in *Escherichia coli* sequence type 744 after meropenem and colistin therapy. *Portugal. Emerg. Infect. Dis.* 23 1419–1421. 10.3201/eid2308.170162 28726622 PMC5547783

[B24] Veldman K. van Essen-Zandbergen A. Rapallini M. Wit B. Heymans R. van Pelt W. (2016). Location of colistin resistance gene *mcr-1* in *Enterobacteriaceae* from livestock and meat. *J. Antimicrob. Chemother.* 71 2340–2342. 10.1093/jac/dkw181 27246233

[B25] Wu C. Wang Y. Shi X. Wang S. Ren H. Shen Z. (2018). Rapid rise of the ESBL and *mcr-1* genes in *Escherichia coli* of chicken origin in China, 2008–2014. *Emerg. Microbes. Infect.* 7:30. 10.1038/s41426-018-0033-1 29535301 PMC5849743

[B26] Wu R. Yi L.-X. Yu L.-F. Wang J. Liu Y. Chen X. (2018). Fitness Advantage of mcr-1–bearing IncI2 and IncX4 plasmids *in vitro*. *Front. Microbiol.* 9:331 10.3389/fmicb.2018.00331 PMC5835064 29535696

[B27] Zweifel C. Schumacher S. Beutin L. Blanco J. Stephan R. (2006). Virulence profiles of Shiga toxin 2e-producing *Escherichia coli* isolated from healthy pig at slaughter. *Vet. Microbiol.* 117 328–332. 10.1016/j.vetmic.2006.06.017 16872761

